# Effect of Ventilation on Physiological Recovery During Midday Naps: A Heart Rate Variability Analysis of Office Workers

**DOI:** 10.3390/s26092716

**Published:** 2026-04-28

**Authors:** Atsushi Yamada, Naoya Morikawa, Emi Yuda

**Affiliations:** 1 Innovation Center for Semiconductor and Digital Future, Mie University, Tsu 514-0102, Mie, Japan; a-yamada@fujioh.com; 2 Graduate School of Engineering, Mie University, Tsu 514-0102, Mie, Japan; 426m532@m.mie-u.ac.jp; 3 Department of Management Science and Technology, School of Engineering, Tohoku University, Sendai 980-8577, Miyagi, Japan

**Keywords:** heart rate variability (HRV), midday nap, autonomic nervous system, parasympathetic reactivation, wearable electrocardiogram (ECG)

## Abstract

Background: In modern office environments, maintaining adequate air quality is essential for cognitive performance and overall well-being. However, the physiological effects of ventilation (CO_2_ control) during short daytime breaks, particularly midday naps, remain insufficiently explored. This study aimed to investigate the impact of ventilation on autonomic nervous system (ANS) activity using heart rate variability (HRV) metrics. Methods: A crossover experiment was conducted with six office workers (mean age: 28 ± 2 years). Two conditions were compared: Condition A (with ventilation/CO_2_ control) and Condition B (without ventilation). The experimental protocol consisted of three phases: Phase 1 (desk work, 11:00–12:00), Phase 2 (nap, 12:00–13:00, including a 20–25 min nap), and Phase 3 (post-nap desk work, 13:00–14:00). HRV indices (SDNN, RMSSD, CVRR, LF, HF, and LF/HF) were calculated from 5-min segments within each phase. Results: A two-way mixed ANOVA revealed a significant main effect of ventilation on the LF/HF ratio during the post-nap phase (*p* = 0.0050, η^2^_p_ = 0.9901), indicating improved autonomic stability upon awakening. Furthermore, a three-way mixed ANOVA (Sex × Order × Condition) showed that pNN50, an index of parasympathetic activity, exhibited significant interactions during the nap phase, including Condition × Sex (*p* = 0.0092) and the three-way interaction (*p* = 0.0333). Significant Order effects were also observed for heart rate (HR) across all phases (*p* < 0.05), suggesting habituation to the experimental environment. Conclusions: These findings indicate that ventilation is a critical environmental factor influencing physiological recovery during midday naps. Optimizing air quality may enhance autonomic regulation and improve the restorative effects of short daytime sleep in office settings. These findings should be interpreted as exploratory due to the small sample size (n = 6). While LF/HF was used as an index of autonomic balance, its physiological interpretation remains debated and should be considered with caution.

## 1. Introduction

Midday naps, or short daytime rest periods, have been widely reported to be effective in alleviating fatigue and maintaining cognitive performance in professional environments. However, much of the existing evaluation of these restorative effects relies on subjective assessments, such as self-reported questionnaires, or behavioral outcomes, including task performance. Objective physiological evaluations based on real-time biological signals remain insufficient to fully characterize the underlying recovery processes.

Heart rate variability (HRV) has been extensively used as a non-invasive index of autonomic nervous system (ANS) activity and provides a quantitative framework for assessing physiological recovery. A substantial body of literature has established the fundamental relationship between sleep and HRV. In particular, previous studies [1,2,3,4,5,6,7,8,9,10] have demonstrated that HRV is strongly modulated by sleep architecture, with non-rapid eye movement (NREM) sleep—especially slow-wave sleep—being associated with enhanced parasympathetic activity, while rapid eye movement (REM) sleep exhibits more variable and often sympathetic-dominant patterns. In addition, circadian rhythms have been shown to significantly influence HRV dynamics, independently of sleep itself, indicating that both sleep stage and biological timing must be considered when interpreting autonomic changes.

Beyond HRV alone, several studies have investigated the relationship between autonomic activity and other physiological signals, such as electroencephalography (EEG), electrooculography (EOG), and respiration [11,12,13,14,15,16]. These multimodal approaches have revealed that HRV indices correlate with neural activity patterns and sleep depth, supporting the validity of HRV as a surrogate marker of sleep-related physiological states. Nevertheless, the absence of direct neurophysiological measurements in many applied studies limits the interpretability of HRV-based findings.

Furthermore, prior research has explored the effects of sleep-related conditions such as short naps, sleep deprivation, and shift work on HRV [17,18,19,20,21,22,23,24]. These studies suggest that even brief naps can enhance parasympathetic activity and improve autonomic balance, whereas sleep deprivation and irregular work schedules tend to disrupt HRV patterns and impair recovery. Despite these insights, the majority of these studies have focused on sleep duration and timing, with comparatively little attention given to environmental factors that may modulate the restorative effects of sleep.

Concurrently, indoor air quality—particularly carbon dioxide (CO_2_) concentration as an indicator of ventilation—has been associated with various cognitive and physiological effects. Elevated CO_2_ levels in poorly ventilated indoor environments have been linked to increased drowsiness, reduced cognitive performance, and impaired decision-making. As modern office buildings become increasingly airtight to improve energy efficiency, concerns regarding the physiological consequences of inadequate ventilation have intensified. However, while the cognitive and behavioral effects of CO_2_ exposure have been studied, its influence on autonomic recovery processes during sleep or short naps remains largely unexplored.

In this study, the ventilation condition was predefined as a primary environmental factor within the experimental design. Therefore, the purpose of this study was to objectively evaluate how differences in ventilation conditions—specifically, well-ventilated (Condition A) versus poorly ventilated (Condition B) environments—affect physiological recovery during and after midday naps. By employing HRV analysis as a sensitive proxy for autonomic nervous system activity, this study aims to quantify the restorative quality of short daytime sleep under varying CO_2_ concentrations. Through this approach, we seek to provide new insights into the interaction between environmental factors and sleep-related recovery, thereby contributing to the optimization of indoor environments for enhancing both health and productivity in office settings.

## 2. Materials and Methods

### 2.1. Ethical Considerations

This study was conducted in accordance with the Declaration of Helsinki and approved by the Ethics Committee of the Graduate School of Engineering, Mie University (Approval Number: 134; Approval Date: 9 October 2025). All participants provided written informed consent after receiving a detailed explanation of the study’s purpose, procedures, and potential risks.

### 2.2. Participants and Experimental Design

Six healthy office workers (mean age: 28 ± 2 years) participated in a randomized crossover trial. To minimize order effects, the subjects were assigned to two sequences: Participants 1, 3, and 5 performed Condition A (Ventilated) followed by Condition B (Non-ventilated), while Participants 2, 4, and 6 followed the reverse order. In this study, the ventilation status was quantified by the indoor CO_2_ concentration. The mean CO_2_ concentration under Condition A (Ventilated) was 600 ± 100 ppm (range: 450–750 ppm), representing a well-ventilated environment. In contrast, under Condition B (Non-ventilated), the mean CO_2_ concentration reached 1600 ± 350 ppm (range: 1200–2200 ppm) due to the limited air exchange in the enclosed space.

### 2.3. Experimental Protocol

**Figure 1:**
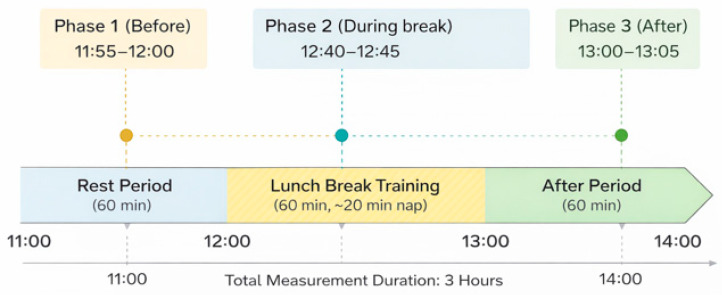
Experimental workflow for ventilation and non-ventilation conditions.

The experiment was conducted between 11:00 and 14:00 and divided into three distinct phases: Phase 1 (Pre-nap, 11:00–12:00): Desk work under the assigned environmental condition. Phase 2 (Nap, 12:00–13:00): A midday break including a 20–25 min nap. Phase 3 (Post-nap, 13:00–14:00): Resumption of desk work to evaluate recovery effects (Figure 1).

Six participants (three female) completed the physiological measurements under two environmental conditions: Condition A (ventilated) and Condition B (non-ventilated). To counterbalance order effects, Participants 1, 3, and 5 performed the experiment in the sequence A → B, whereas Participants 2, 4, and 6 completed the sequence B → A. All participants underwent both conditions, enabling within-subject comparisons of physiological responses. It should be noted that while heart rate (HR) data alone are insufficient to definitively determine sleep stages or the onset of sleep, they can be used to estimate the possibility of sleep. Specifically, a low and stable HR trend during the scheduled nap period suggests a high probability of sleep, although the accuracy of this estimation remains limited without concurrent neurophysiological measurements such as EEG.

**Figure 2:**
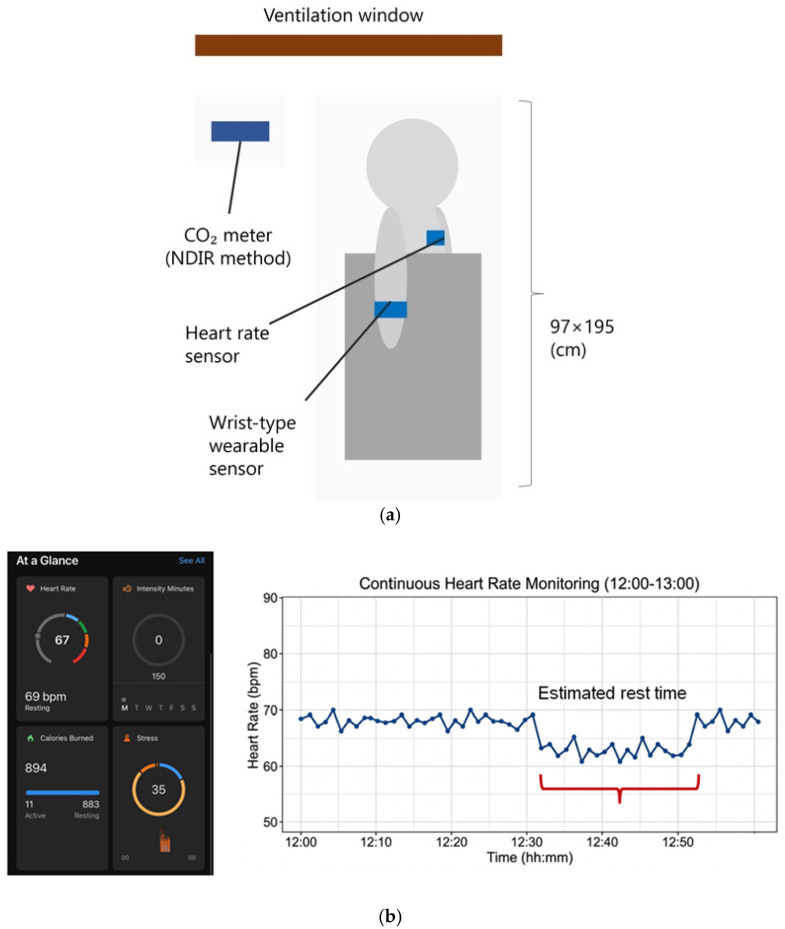
Experimental environment setup.

The experimental environment was controlled to ensure consistent physiological measurements across participants (Figure 2a). A chest-mounted heart rate sensor was placed on the left side of the chest, and a wristband-type wearable sensor was worn on the right arm to capture additional physiological signals. The room temperature was maintained at 24 °C ± 2 °C, and the carbon dioxide concentration was continuously monitored using a CO_2_ meter to ensure stable air quality throughout the experiment. Figure 2b illustrates an example of real-time heart rate (HR) monitoring using a wrist-worn wearable sensor. The HR data, which can be monitored in real-time via the sensor, serves as a reliable proxy for estimating nap duration and sleep states. While individual variations were observed in sleep onset and wake-up times, the data clearly indicated a physiological transition to a sleep state. Specifically, for this subject, a sustained decrease in HR was observed for approximately 20 min between 12:30 and 12:50, from which the nap period was estimated (Figure 2b).

### 2.4. Physiological and Environmental Monitoring

Physiological data were continuously monitored using wearable sensors. ECG and HRV were recorded using a chest-strap type wearable sensor (myBeat, Union Tool Co., Tokyo, Japan) to capture R-R intervals with high temporal resolution. R-peak detection was performed using an automated algorithm implemented in the wearable ECG system, the reliability of which has been validated in previous studies. For secondary physiological parameters and confirmation of nap duration consistency, a wrist-worn smart device (vívosmart 5, Garmin Ltd., Olathe, KS, USA) was employed.

Environmental conditions, including CO_2_ concentration, temperature, and humidity, were monitored using a high-precision data logger, TR-76Ui (T&D Corporation, Tokyo, Japan). The device features a wide measurement range for CO_2_ from 0 to 9999 ppm, with an accuracy of ±50 ppm ±5% of the reading. It also measures temperature (0 to 55 °C, ±0.5 °C) and humidity (10 to 95% RH, ±5% RH at 25 °C, 50% RH). The TR-76Ui has a data logging capacity of 8000 readings per channel, with selectable recording intervals ranging from 1 s to 60 min. For study management, the T&D Recorder for Windows software was utilized.

### 2.5. Data Analysis

Prior to the calculation of HRV indices, R-R intervals were preprocessed to ensure data quality. Ectopic beats and artifacts exceeding ±20% of the local mean were removed, followed by a manual visual inspection. Any missing values resulting from artifact removal were interpolated using cubic spline interpolation. HRV indices were calculated for 5-min segments within each phase. Time-domain and frequency-domain metrics included SDNN, RMSSD, CVRR, NN50, pNN50, AC, DC, average heart rate (HRAve), LF, HF, VLF, LF/HF, and normalized components (LFnorm, HFnorm). The primary endpoints of this study were predefined as (1) the LF/HF ratio during the post-nap phase (Phase 3) and (2) pNN50 during the nap phase (Phase 2). All other HRV parameters and indices were considered exploratory. Statistical analysis was performed to compare the physiological impact of the two ventilation conditions. Mauchly’s test of sphericity was conducted to assess the assumption of sphericity, and Greenhouse–Geisser correction was applied where appropriate. Homogeneity of variance was assessed using Levene’s test. The calculated HRV indices were categorized and defined as follows (Table 1).

Statistical analysis was conducted using a within-subject (repeated-measures) design, in which all participants completed both ventilation conditions: Condition A (with ventilation) and Condition B (without ventilation). To minimize potential bias due to experimental sequencing, the order of conditions was counterbalanced across participants. Specifically, participants 1, 3, and 5 underwent the protocol in the order A→B, whereas participants 2, 4, and 6 followed the order B→A. To evaluate the physiological effects of ventilation while accounting for potential order effects, a two-way mixed-design ANOVA was first conducted. In this model, Condition (A vs. B) was treated as a within-subject factor, and Order (A→B vs. B→A) was treated as a between-subject factor. Subsequently, to further examine potential sex-related differences, a three-way repeated-measures ANOVA model was applied, including Condition (within-subject), Sex (male vs. female), and Order (between-subject) as factors. Given the small sample size (n = 6), statistical significance alone may not adequately reflect the magnitude of the observed effects. Therefore, effect sizes (partial eta squared, η^2^_p_) were calculated for all ANOVA results to provide a more comprehensive interpretation. Furthermore, given the exploratory nature of this study, no strict correction for multiple comparisons was applied to the various HRV indices analyzed. As part of the analytical workflow, the following steps were performed:

1. Calculation of within-subject differences (A − B) for each HRV index.
2. Assessment of normality of the difference distributions (Shapiro–Wilk test).
3. Evaluation of order effects and interaction effects using mixed-design ANOVA.
4. Application of the three-factor repeated-measures ANOVA including sex.

All statistical analyses were conducted using a significance level of *p* < 0.05.

## 3. Results

### 3.1. Normality and Baseline Characteristics

The Shapiro-Wilk test confirmed that the difference scores (Diff) for all heart rate variability (HRV) metrics followed a normal distribution (*p* > 0.05, W ≈ 1.0). Consequently, parametric statistical methods were employed for all subsequent analyses (Table 2). Furthermore, environmental monitoring data confirmed that CO_2_ levels were consistently higher in the non-ventilated condition (Condition B) compared to the ventilated condition (Condition A), establishing a clear and significant environmental difference between the two experimental settings throughout all phases.

### 3.2. Two-Way Mixed ANOVA: Order and Condition Effects

Across all phases (Pre-, During-, and Post-Nap), a significant main effect of Order was observed for heart rate (HR) (Pre: *p* = 0.0197; During: *p* = 0.0257; Post: *p* = 0.0482), indicating an experimental sequence effect. Regarding the Condition effect (Ventilation vs. Non-Ventilation), a significant difference was observed in the LF/HF ratio during the Post-Nap phase (*p* = 0.0387, η^2^_p_ = 0.6969). Additionally, a significant Interaction (Order × Condition) was found for VLF in the Post-Nap phase (*p* = 0.0405) (Table 3).

### 3.3. Three-Way Mixed ANOVA: Sex, Order, and Condition Interactions

The inclusion of Sex as a factor revealed complex interactions: Pre-Nap: pNN50 showed a significant effect for Condition (*p* = 0.0324) and a Sex × Condition interaction (*p* = 0.0299). During Nap: pNN50 exhibited highly significant interactions for Cond × Sex (*p* = 0.0092), Cond × Order (*p* = 0.0394), and the three-way Sex × Order × Condition interaction (*p* = 0.0333). Post-Nap: LF/HF demonstrated a highly significant Condition effect (*p* = 0.0050) and a three-way interaction (*p* = 0.0062). SDNN also showed a significant Sex × Condition interaction (*p* = 0.0375) (Table 4).

Mean_Diff and SD_Diff represent the mean and standard deviation of the within-subject differences between Condition A (Ventilated) and Condition B (Non-ventilated). W denotes the Shapiro–Wilk test statistic. All metrics satisfied the assumption of normality (*p* > 0.05), justifying the use of parametric statistical tests.

### 3.4. Individual Variations in Autonomic Responses

**Figure 3:**
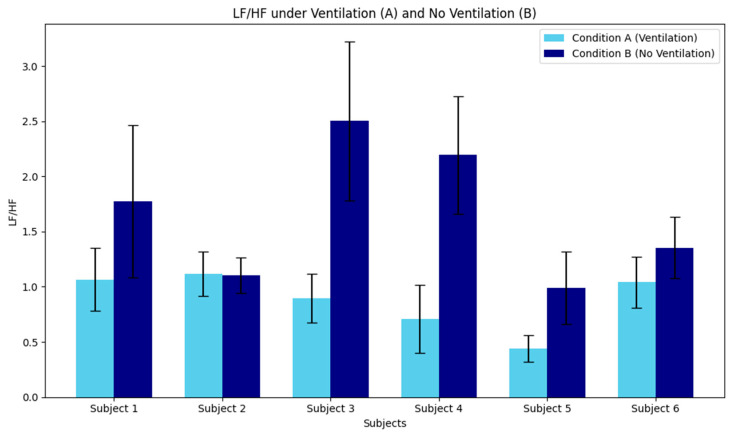
Individual responses of LF/HF ratio under different ventilation conditions during nap phase.

**Figure 4:**
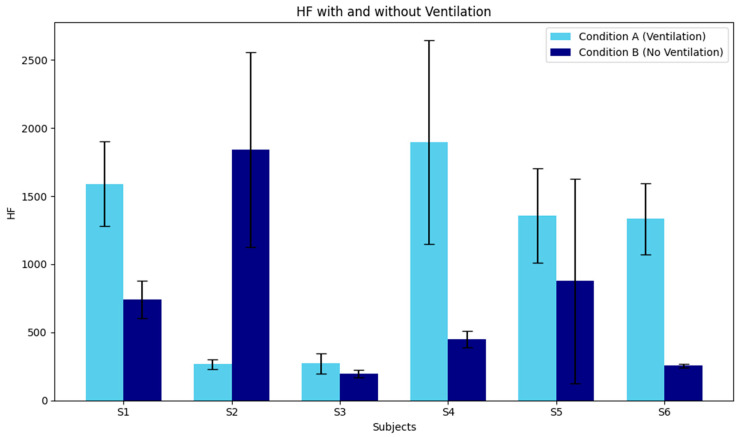
Individual responses of HF ratio under different ventilation conditions during nap phase.

The individual physiological responses to ventilation conditions during the during nap recovery phase are illustrated in Figure 3 and Figure 4. Regarding the LF/HF ratio (Figure 3), although a significant main effect of Condition was identified in the group-level ANOVA, substantial individual variability was observed. Most subjects exhibited a higher LF/HF ratio in Condition B (Non-ventilated) compared to Condition A (Ventilated), suggesting that inadequate ventilation may consistently shift the autonomic balance toward sympathetic dominance across different individuals. In contrast, the HF component (Figure 4), an index of parasympathetic activity, showed even more pronounced inter-individual differences. While subjects such as S1, S4, S5, and S6 demonstrated higher HF power under Condition A—indicating superior physiological relaxation in a well-ventilated environment—other subjects showed divergent patterns. This highlights that while environmental air quality significantly modulates autonomic recovery, the magnitude and direction of the effect on parasympathetic reactivation can vary depending on individual physiological characteristics. These results underscore the necessity of considering individual variability when evaluating the impact of indoor air quality on human health and restorative sleep.

This bar chart illustrates the mean and standard deviation (SD) of the LF/HF ratio for each of the six subjects under the two experimental conditions. The LF/HF ratio serves as an indicator of autonomic balance, specifically reflecting the ratio of sympathetic to parasympathetic nervous system activity. Vertical Axis (Y-axis): LF/HF ratio (unitless). A higher value typically indicates higher sympathetic dominance or relative parasympathetic withdrawal. Horizontal Axis (X-axis): Individual subjects (Subject 1 to Subject 6). Condition A (Sky Blue): Ventilated environment (with air exchange). Condition B (Navy): Non-ventilated environment (closed space with elevated CO_2_ levels). Observations: Across the majority of subjects, Condition B (No Ventilation) exhibited a higher mean LF/HF ratio compared to Condition A (Ventilation). This trend suggests that inadequate ventilation during or after a midday nap may lead to increased sympathetic activation or delayed autonomic recovery. The error bars represent the standard deviation, indicating the physiological variability within each 5-min analysis segment for each participant.

This chart presents the mean and standard deviation (SD) of the HF component (ms^2^) for each of the six subjects. The HF component is widely recognized as a primary indicator of parasympathetic nervous system activity, often associated with relaxation and restorative processes during and after sleep. Vertical Axis (Y-axis): HF power spectral density (ms^2^). Higher values generally reflect enhanced parasympathetic activity or better physiological relaxation. Horizontal Axis (X-axis): Individual subjects (S1 to S6). Condition A (Sky Blue): Ventilated environment (Condition A). Condition B (Navy): Non-ventilated environment (Condition B). Observations: The data revealed considerable individual variability in the HF response to ventilation. For several subjects (e.g., S1, S4, S5, and S6), Condition A (Ventilation) demonstrated higher HF power compared to Condition B, suggesting that a well-ventilated environment may facilitate superior parasympathetic activation during the recovery period. Conversely, the substantial SD observed in certain participants (e.g., S4 and S2 under specific conditions) reflects the dynamic nature of autonomic fluctuations within the analysis segments.

## 4. Discussion

The present study investigated the impact of ventilation on physiological recovery during midday naps among office workers using HRV analysis. Our findings suggest that while the physical act of napping provides recovery, the quality of this recovery—specifically autonomic nervous system (ANS) modulation—is significantly influenced by environmental ventilation and individual factors such as biological sex. However, given the exploratory nature of this study, the findings should be regarded as preliminary and hypothesis-generating rather than confirmatory.

### 4.1. Impact of Ventilation on Autonomic Balance

The most notable finding was the significant effect of the ventilation condition on the LF/HF ratio during the post-nap phase. While the observed effect size was large (η^2^_p_ = 0.6969), it must be noted that extremely large effect sizes in this study may be inflated due to the small sample size (n = 6) and should be interpreted with caution. Furthermore, although LF/HF is often interpreted as an index of sympathovagal balance, this interpretation remains controversial. LF components reflect mixed autonomic influences rather than pure sympathetic activity, and the ratio should not be considered as a direct physiological measure of sympathetic outflow. Nevertheless, the results are consistent with previous studies indicating that environmental factors such as CO_2_ concentration can modulate cardiac autonomic regulation. The observed reduction in LF/HF under ventilated conditions may facilitate a smoother transition from sleep to wakefulness, potentially mitigating sleep inertia, though HRV indices themselves are influenced by multiple factors including respiration, posture, and measurement conditions, which limits their interpretability in real-world environments.

### 4.2. Sex-Specific Physiological Responses

The three-way ANOVA revealed that Sex is a critical moderator of the physiological response to ventilation. The significant interactions in pNN50—a marker of parasympathetic activity—suggest that females and males may respond differently to air quality changes during sleep. This finding aligns with literature reporting sex differences and age-related variations in HRV dynamics [3,25,26,27]. Recent work has proposed HRV as a digital biomarker reflecting individual physiological variability [27]. The significant Sex × Condition interactions in SDNN and pNN50 during the post-nap phase indicate that ventilation may enhance parasympathetic reactivation more effectively in specific groups. This highlights the potential need for personalized environmental control, particularly considering sex differences in thermoregulation and respiratory sensitivity.

### 4.3. Influence of Experimental Order and Limitations in Sleep Assessment

A persistent significant effect of Order on heart rate (HR) suggests a “first-visit effect”. However, the robustness of ventilation effects (LF/HF and pNN50) indicates that environmental modulation operates independently of habituation. A primary limitation of this study is that sleep stages were not objectively assessed using EEG or polysomnography. Consequently, nap periods were estimated based on protocol timing and heart rate trends, which may not fully capture the nuances of sleep architecture. While HRV is known to reflect states like insomnia or sleep disorders [28,29,30], the lack of direct neurophysiological validation in this study necessitates a cautious interpretation of the “nap” quality itself.

### 4.4. Implications and Future Directions

These results suggest that integrating smart ventilation systems in office rest areas could optimize the restorative value of midday naps by maintaining parasympathetic dominance. Recent advances in physiological monitoring, such as pulse transit time (PTT) [16] and sleep prediction models [31], support the potential for multimodal optimization of sleep environments. While these findings suggest potential applications, further large-scale studies are required before clinical implications—such as the mitigation of stroke or cognitive decline [32]—can be established. Future research should incorporate larger sample sizes, objective sleep stage monitoring, and cognitive performance measures to link HRV changes with functional outcomes and long-term health benefits.

## 5. Conclusions

This study provides preliminary evidence that environmental ventilation may influence autonomic nervous system (ANS) recovery during and after midday naps among office workers. Based on the observed data, the following points are summarized:

1. Potential for Optimized Post-Nap Recovery: The observed effect of ventilation on the LF/HF ratio during the post-nap phase suggests that controlled air quality may play a role in stabilizing autonomic balance upon waking. This implies that improved ventilation could facilitate physiological recovery, though the practical impact on mitigating sleep inertia requires further functional validation.
2. Context-Dependent Parasympathetic Modulation: The significant interactions involving pNN50 during the nap phase indicate that the effects of ventilation are not uniform. The impact on parasympathetic reactivation appears to be moderated by individual factors, such as biological sex and experimental order.

In conclusion, while ventilation appears to be a relevant environmental factor interacting with individual physiological traits, these findings should be interpreted cautiously due to the small sample size and exploratory nature of the analysis. These results serve as a basis for future, larger-scale studies to validate whether person-centric ventilation systems can effectively enhance the restorative value of short daytime breaks in office environments.

## Figures and Tables

**Table 1:** Definition and physiological significance of calculated HRV indices.

Category	Index (Unit)	Definition/Calculation Method	Physiological Significance (Interpretation)
Time-Domain	SDNN (ms)	Standard deviation of all NN intervals	Overall ANS activity and total variability
	RMSSD (ms)	Root mean square of successive differences	Parasympathetic activity (vagal tone)
	CVRR (%)	SDNN/Mean HR	Normalized variability index (coefficient of variation)
	NN50 (count)	Number of successive NN intervals > 50 ms	Intensity of parasympathetic modulation
	pNN50 (%)	Percentage of NN50 within the total NN intervals	Precision index for parasympathetic dominance
Frequency-Domain	HF (ms^2^)	High frequency (0.15–0.40 Hz)	Parasympathetic (vagal) activity; respiratory influence
	LF (ms^2^)	Low frequency (0.04–0.15 Hz)	Baroreflex activity; sympathetic and parasympathetic mix
	VLF (ms^2^)	Very low Frequency (0.0033–0.04 Hz)	Thermoregulation, hormonal, and metabolic processes
	Total Power	Sum of VLF, LF, and HF	Total autonomic regulatory capacity
	LF/HF	Ratio of LF to HF power	Sympathovagal balance (sympathetic/parasympathetic)
	LF_norm_/HF_norm_	Ratio of LF or HF to (Total Power—VLF)	Relative contribution of sympathetic or parasympathetic
Non-Linear/Others	AC (ms)	Acceleration capacity (via PRSA)	Regulatory capacity of the sympathetic nervous system
	DC (ms)	Deceleration capacity (via PRSA)	Regulatory capacity of the parasympathetic nervous system
	HR_Ave_ (bpm)	Average heart rate	Baseline circulatory and metabolic reference

**Table 2:** Descriptive statistics and Shapiro–Wilk normality test for HRV metric differences (Condition A − Condition B).

Phase	Metric	Mean_Diff	SD_Diff	W	*p*-Value
Pre-Nap	HR (bpm)	−0.878	9.966	0.910	0.438
	SDNN (ms)	0.667	17.227	0.866	0.211
	pNN50 (%)	−13.328	16.958	0.920	0.506
	VLF (ms^2^)	260.181	299.590	0.949	0.728
	LF (ms^2^)	−119.112	456.382	0.905	0.407
	HF (ms^2^)	−372.984	498.400	0.919	0.500
	LF/HF	1.314	2.349	0.849	0.155
During Nap	HR (bpm)	−1.836	11.472	0.907	0.419
	SDNN (ms)	−2.798	27.963	0.872	0.235
	pNN50 (%)	−12.261	29.376	0.858	0.183
	VLF (ms^2^)	4.439	981.199	0.964	0.847
	LF (ms^2^)	93.100	894.769	0.931	0.588
	HF (ms^2^)	−392.215	1074.671	0.883	0.284
	LF/HF	0.775	0.646	0.917	0.486
Post-Nap	HR (bpm)	−1.747	8.676	0.849	0.155
	SDNN (ms)	1.354	15.846	0.954	0.770
	pNN50 (%)	−12.360	19.650	0.932	0.599
	VLF (ms^2^)	2203.118	3526.183	0.967	0.871
	LF (ms^2^)	−123.255	638.589	0.923	0.524
	HF (ms^2^)	−471.495	536.240	0.828	0.103
	LF/HF	0.677	0.495	0.800	0.059

**Table 3:** Results of 2-way mixed ANOVA for HRV metrics.

Phase	Metric	Source	F	df_1_	df_2_	*p*	η^2^_p_
							
Pre-Nap	HR	Order	14.1923	1	4	0.0197 *	0.7801
		Condition	0.0383	1	4	0.8544	0.0095
		Interaction	0.1135	1	4	0.7531	0.0276
	SDNN	Order	3.8547	1	4	0.1211	0.4907
		Condition	0.0072	1	4	0.9364	0.0018
		Interaction	0.0070	1	4	0.9372	0.0018
	pNN50	Order	1.8972	1	4	0.2404	0.3217
		Condition	2.9653	1	4	0.1602	0.4257
		Interaction	0.0003	1	4	0.9860	0.0001
	VLF	Order	5.1170	1	4	0.0865	0.5613
		Condition	3.6490	1	4	0.1287	0.4771
		Interaction	0.0317	1	4	0.8673	0.0079
	LF	Order	4.1625	1	4	0.1109	0.5100
		Condition	0.4269	1	4	0.5492	0.0964
		Interaction	1.2228	1	4	0.3308	0.2341
	HF	Order	0.6984	1	4	0.4503	0.1486
		Condition	2.9378	1	4	0.1617	0.4235
		Interaction	0.3714	1	4	0.5752	0.0850
	LF/HF	Order	3.1925	1	4	0.1485	0.4439
		Condition	1.8693	1	4	0.2433	0.3185
		Interaction	0.9803	1	4	0.3782	0.1968
During Nap	HR	Order	12.0015	1	4	0.0257 *	0.7500
		Condition	0.1254	1	4	0.7412	0.0304
		Interaction	0.0785	1	4	0.7932	0.0192
	SDNN	Order	1.0709	1	4	0.3592	0.2112
		Condition	0.0497	1	4	0.8344	0.0123
		Interaction	0.1391	1	4	0.7281	0.0336
	pNN50	Order	2.1116	1	4	0.2198	0.3455
		Condition	0.8501	1	4	0.4087	0.1753
		Interaction	0.0667	1	4	0.8090	0.0164
	VLF	Order	0.0126	1	4	0.9161	0.0031
		Condition	0.0001	1	4	0.9921	0.0000
		Interaction	0.4923	1	4	0.5216	0.1096
	LF	Order	0.2381	1	4	0.6511	0.0562
		Condition	0.0521	1	4	0.8307	0.0129
		Interaction	0.0082	1	4	0.9323	0.0020
	HF	Order	0.2707	1	4	0.6303	0.0634
		Condition	0.6432	1	4	0.4675	0.1385
		Interaction	0.0238	1	4	0.8848	0.0059
	LF/HF	Order	0.0063	1	4	0.9404	0.0016
		Condition	7.5929	1	4	0.0511	0.6550
		Interaction	0.4015	1	4	0.5607	0.0912
Post-Nap	HR	Order	7.9063	1	4	0.0482 *	0.6640
		Condition	0.1973	1	4	0.6799	0.0470
		Interaction	0.0527	1	4	0.8297	0.0130
	SDNN	Order	2.0639	1	4	0.2242	0.3404
		Condition	0.0390	1	4	0.8530	0.0097
		Interaction	0.4509	1	4	0.5387	0.1013
	pNN50	Order	0.7966	1	4	0.4225	0.1661
		Condition	1.8993	1	4	0.2402	0.3220
		Interaction	0.0006	1	4	0.9815	0.0002
	VLF	Order	5.4388	1	4	0.0801	0.5762
		Condition	6.0510	1	4	0.0697	0.6020
		Interaction	8.9175	1	4	0.0405 *	0.6903
	LF	Order	1.7570	1	4	0.2556	0.3052
		Condition	0.1801	1	4	0.6931	0.0431
		Interaction	0.0293	1	4	0.8723	0.0073
	HF	Order	0.4720	1	4	0.5298	0.1055
		Condition	3.7113	1	4	0.1263	0.4813
		Interaction	0.0005	1	4	0.9828	0.0001
	LF/HF	Order	0.2071	1	4	0.6727	0.0492
		Condition	9.1952	1	4	0.0387 *	0.6969
		Interaction	0.1040	1	4	0.7633	0.0253

Order denotes the between-subjects factor; Condition denotes the within-subjects factor; Interaction refers to the Order × Condition effect. η^2^_p_ indicates partial eta squared (effect size). *p* < 0.05 indicates statistical significance. Significant Order effects were observed for HR across all phases, suggesting that the sequence of trials influenced heart rate irrespective of condition. A significant Interaction effect was observed for VLF in the post-nap phase (*p* = 0.0405), and a significant Condition effect was observed for LF/HF in the post-nap phase (*p* = 0.0387). * *p* < 0.05: Indicates statistical significance.

**Table 4:** Results of 3-way mixed ANOVA (Sex × Order × Condition).

Phase	Metric	Source	F	df_1_	df_2_	*p*	η^2^_p_
Pre-Nap	HR	Sex	0.6982	1	2	0.4913	0.2588
		Order	17.5401	1	2	0.0526	0.8976
		Sex × Order	2.1482	1	2	0.2804	0.5179
		Condition	0.0050	1	2	0.9501	0.0025
		Cond × Sex	1.2142	1	2	0.3854	0.3778
		Cond × Order	0.0034	1	2	0.9585	0.0017
		Cond × Sex × Order	0.5620	1	2	0.5317	0.2193
	SDNN	(Significant: None)					
		Cond × Sex	7.5741	1	2	0.1106	0.7911
	pNN50	Condition	29.3535	1	2	0.0324 *	0.9362
		Cond × Sex	31.9464	1	2	0.0299 *	0.9411
	VLF	Order	10.1127	1	2	0.0863	0.8349
	HF	Condition	7.3504	1	2	0.1134	0.7861
		Cond × Sex	10.4510	1	2	0.0838	0.8394
During Nap	HR	Order	12.1094	1	2	0.0736	0.8583
	pNN50	Condition	11.0657	1	2	0.0797	0.8469
		Cond × Sex	107.5387	1	2	0.0092 *	0.9817
		Cond × Order	23.8821	1	2	0.0394 *	0.9227
		Cond × Sex × Order	28.5522	1	2	0.0333 *	0.9345
	LF/HF	Sex × Order	10.5356	1	2	0.0832	0.8405
Post-Nap	HR	Order	6.0511	1	2	0.1331	0.7516
	SDNN	Cond × Sex	25.1686	1	2	0.0375 *	0.9264
		Cond × Order	11.0642	1	2	0.0797	0.8469
	pNN50	Condition	17.8239	1	2	0.0518	0.8991
		Cond × Sex	44.9697	1	2	0.0215 *	0.9574
	VLF	Interaction (all)	8.9175	1	2	0.0405 *	0.6903
	LF/HF	Condition	199.5860	1	2	0.0050 *	0.9901
		Cond × Sex × Order	160.7151	1	2	0.0062 *	0.9877

* *p* < 0.05: Indicates statistical significance. Key Findings; Pre-Nap: pNN50 showed a significant effect for Condition (*p* = 0.0324) and a Sex–Condition interaction (*p* = 0.0299). During Nap: Significant interactions were found in pNN50 for Cond × Sex (*p* = 0.0092), Cond × Order (*p* = 0.0394), and the three-way interaction (*p* = 0.0333). Post-Nap: LF/HF demonstrated a highly significant Condition effect (*p* = 0.0050) and a three-way interaction (*p* = 0.0062). SDNN and pNN50 also showed significant Sex–Condition interactions.

## Data Availability

The data presented in this study are available on request from the corresponding author for academic purposes. The data are not publicly available due to privacy and ethical restrictions.
